# Assistance to patients eligible for palliative care: the view of professionals from an Intensive Care Unit

**DOI:** 10.1590/1980-220X-REEUSP-2021-0429en

**Published:** 2022-06-01

**Authors:** Matheus Rodrigues Martins, Juliana da Silva Oliveira, Alexandre Ernesto Silva, Rudval Souza da Silva, Tatiane Oliveira de Souza Constâncio, Sheylla Nayara Sales Vieira

**Affiliations:** 1 Universidade Estadual do Sudoeste da Bahia, Programa de Residência Multiprofissional em Urgência e Emergência, Jequié, BA, Brazil.; 2 Universidade Estadual do Sudoeste da Bahia, Departamento de Saúde II, Jequié, BA, Brazil.; 3 Universidade Federal de São João Del-Rei, Enfermagem Fundamental, Divinópolis, MG, Brazil.; 4 Universidade do Estado da Bahia, Campus VII, Senhor do Bonfim, BA, Brazil.; 5 Universidade Estadual do Sudoeste da Bahia, Programa de Pós-Graduação em Enfermagem e Saúde, Jequié, BA, Brazil.

**Keywords:** Palliative care, Quality Indicators, Health Care, Intensive care units, Patient care team, Health manager, Patient safety, Cuidados paliativos, Indicadores de calidad de la atención de salud, Unidades de cuidados intensivos, Grupo de atención al paciente, Gestor de salud, Seguridad del paciente, Cuidados paliativos, Indicadores de qualidade em assistência à saúde, Unidade de terapia intensiva, Equipe de Assistência ao Paciente, Gestor de saúde, Segurança do paciente

## Abstract

**Objective:**

to understand the perception of the multiprofessional team about the quality of health care provided to patients in palliative care in the Intensive Care Unit.

**Method:**

qualitative study, anchored to Donabedian’s theoretical framework, through semi-structured interviews with 35 professionals working in the Intensive Care Unit. For data analysis, the Content Analysis technique was used.

**Results:**

three categories were pre-established: structure, process, and outcome, from which five subcategories emerged: *Deficit* in terms of numbers of workers and professional qualification; Ambience and palliative care; (In)existence of assistance based on the principles of palliative care; Failures in communication and in the interdisciplinary approach and Repercussions of (lack of) assistance.

**Conclusion:**

the study allowed understanding the institutional weaknesses for the operationalization of care provided to patients eligible for palliative care in the Intensive Care Unit setting. Thus, for this philosophy of care to be propagated, the co-participation of managers, professionals, patients, and family members is required, since these gaps cannot be filled without collective involvement.

## RESUMO

**Objetivo:**

compreender a percepção da equipe multiprofissional sobre a qualidade da assistência à saúde prestada a pacientes em cuidados paliativos em Unidade de Terapia Intensiva.

**Método:**

estudo qualitativo, ancorado no referencial teórico de Donabedian, por meio de entrevistas semiestruturadas realizadas com 35 profissionais que atuam em Unidade de Terapia Intensiva. Para análise dos dados, utilizou-se a técnica de Análise de Conteúdo.

**Resultados:**

foram pré-estabelecidas três categorias: estrutura, processo e resultado, das quais emergiram cinco subcategorias: *Déficit* no quantitativo e na qualificação profissional; Ambiência e cuidados paliativos; (In)existência de uma assistência pautada nos princípios dos cuidados paliativos; Falhas na comunicação e na abordagem interdisciplinar; e Repercussões da (des)assistência.

**Conclusão:**

o estudo permitiu compreender as fragilidades institucionais para a operacionalização da assistência dispensada aos pacientes elegíveis para cuidados paliativos no cenário da Unidade de Terapia Intensiva. Dessa forma, para que essa filosofia de cuidado seja propagada, necessita-se da coparticipação dos gestores, profissionais, pacientes e familiares, uma vez que essas lacunas não conseguem ser supridas sem que haja um envolvimento coletivo.

## RESUMEN

**Objetivo:**

comprender la percepción del equipo multiprofesional sobre la calidad de la asistencia a la salud destinada a pacientes en cuidados paliativos en UCI.

**Método:**

estudio cualitativo, basado en el referencial teórico de Donabedian, por medio de entrevistas semiestructuradas realizadas con 35 profesionales que actúan en Unidad de Cuidados Intensivos. Para análisis de los datos, se utilizó la técnica de Análisis de Contenido.

**Resultados:**

Fueron inicialmente establecidas tres clases: estructura, proceso y resultado, de las cuales emergieron cinco subclases: *Déficit* en el cuantitativo y en la calificación profesional; Ambiencia y cuidados paliativos; Inexistencia de una asistencia pautada en los principios de los cuidados paliativos; Fallos en la comunicación y en el abordaje interdisciplinar y repercusiones de la desasistencia.

**Conclusión:**

El estudio nos llevó a comprender las debilidades institucionales para poner en funcionamiento la asistencia dispensada a los pacientes elegibles para cuidados paliativos en el escenario de la UCI. Así, para que esa filosofía de cuidado sea propagada, se necesita la coparticipación de los gestores, profesionales, pacientes y familiares, una vez que esas debilidades no lograrán ser resueltas sin que haya una participación colectiva.

## INTRODUCTION

The worldwide discussion on the applicability of Palliative Care (PC) has been based on the context of the demographic and epidemiological transition experienced in recent decades, along with population aging and the increase in chronic diseases. This fact has directly implicated health care practices, highlighting the need for specialized care that meets the demands of this new epidemiological profile^(1)^.

Therefore, PC is presented as qualified assistance, capable of subsidizing actions that provide greater comfort to the patient and their families. According to the World Health Organization (WHO)^(2)^, PC is conceptualized as comprehensive health care practices, promoted by a multiprofessional team, aimed at patients with life-threatening diseases, based on actions aiming at conditions for the individual’s overall well-being, through the management of symptoms and signs associated with physical, spiritual, psychosocial impairments and an irreproachable assessment for pain prevention and relief.

It is estimated that, each year, about 40 million people need this type of care, including patients in the initial stage of the disease, and about 20 million people lack this approach at the end of life. Of the latter, 90% are diagnosed with some type of chronic non-communicable disease. The most prevalent diseases are related to cardiovascular disorders, neoplasms, chronic obstructive pulmonary diseases, and diabetes, which generate greater functional impairment and dependence^(3)^.

Moreover, patients with the same epidemiological profile have high rates of admissions to Intensive Care Units (ICU), and it is essential to institute measures to improve quality of life, free from unnecessary treatments that prolong suffering^(4)^. Nevertheless, it is observed that the care model adopted in ICUs is generally based on biomedical, curative, and technologically dense principles that do not always allow care centered on basic human needs, well-being, encouragement of self-care, and on the individual’s autonomy^(5,6)^.

However, for this care to be properly offered, it has to be provided by a multidisciplinary team, through a holistic approach. This way, prevention and early identification of suffering in all fields affecting human dignity are allowed^(3)^.

However, most health professionals and managers still associate PC with care aimed only at patients with neoplasms and at the end of their lives, not covering other clinical conditions and the initial period of illness. Such unawareness directly interferes with the planning and quality of care provided^(7)^, given that the lack of knowledge implies disorganized care practices with no scientific evidence, making patients vulnerable to the occurrence of adverse events, leading to prolonged hospitalization and, consequently, increased hospital costs.

Thus, the use of assessment tools that ensure quality of care provided to this public is required. In this context, Donabedian proposes indicators that guide the way to quality care, formed by the triad: structure, process, and outcome^(8)^. The *structure* summarizes the necessary conditions for the occurrence of quality care; the *process* is related to the form of care to be performed, and the *outcome* corresponds to the patient’s response to the care provided^(8)^.

Through the evaluation of these criteria, the level of quality achieved can be measured, strengths and weaknesses can be evaluated, and strategies to correct and improve the unsatisfactory aspects can be sought. Furthermore, quality indicators are recognized as indispensable management tools for the implementation of good practices in the hospital environment, directly guiding health professionals’ decision-making towards evidence-based practice^(9)^.

This study is justified by the need to broadly understand the institutional characteristics influencing the quality of care provided to patients in palliative care, since health units that do not have specialized teams for this purpose and in which PC is not incorporated as a care philosophy present barriers that compromise the dissemination of this practice, such as the *deficit* in professional training, the lack of organization of the work process, the applicability of institutional policies and protocols, as well as the scarcity of bioethical debates^(10)^, which can lead to practices of therapeutic obstinacy and incipient prevention/control of symptoms and signs arising from the illness process.

Thus, through this evaluation process, the provision of comprehensive care covering the patient’s multiple dimensions in all stages of illness becomes possible. This study findings are expected to contribute to the promotion of a discussion on this health area subject, especially for managers and multiprofessional teams working in ICUs, promoting a reflection on the necessary conditions for the quality of care provided to this public. In view of these observations, the following objective was formulated: to understand the perception of the multiprofessional team about the quality of health care provided to CP patients in the ICU.

## METHOD

### Design of Study

This is a descriptive, exploratory study, with a qualitative approach, anchored to the theoretical framework of Avedis Donabedian^(8)^, based on the *Consolidated Criteria For Reporting Qualitative Research* (COREQ)^(11)^.

### Local

As a research setting, the three Adult ICUs of a public tertiary hospital, located in the city of Jequié, Bahia, Brazil, were delimited. The aforementioned nosocomium is considered a state reference unit, serving a population of 27 municipalities, covering the specialties of internal medicine, surgical clinic, pediatrics, psychiatry, and intensive care. However, it does not have a unit and/or team specialized in palliative care, despite the use of the term “palliative care” to define a therapeutic approach to patients in the ICU setting.

### Population

The participants of this research were members of the multidisciplinary team consisting of nurses, nursing technicians, physiotherapists, pharmacists, physicians, psychologists, and nutritionists who offered care to PC patients in the ICUs of that hospital, selected through the non-probabilistic convenience sampling technique.

### Selection Criteria

The inclusion criteria were voluntary participants, of both sexes, with at least five months of care work in the ICU, belonging to the professional categories of nursing (nurses and nursing technicians), physiotherapy, pharmacy, medicine, psychology, and nutrition. The exclusion criteria covered those professionals who were on vacation, on sick leave, long paid leaves, or maternity leave during the data collection period. The number of participants was defined by the criterion of saturation of the answers, being represented by the lack of new data that contributed significantly to the study^(12)^.

### Data Collection

Data were collected by the first author, a nurse, in a reserved place in the hospital unit, from September to December 2020, with times previously agreed between the researcher and the participants. For the collection of information, an instrument with objective questions was used, referring to the sociodemographic aspects to characterize the participants and a semi- structured script for interviews, with guiding questions, based on Donabedian’s triad^(8)^.

The instrument was previously subjected to a pilot study with the participation of 13 multiprofessional health residents, who worked in emergency and ICU departments. After its application, questions that were difficult to understand by the participants were reformulated, and questions that were not related to aspects of the Donabedian triad were excluded^(8)^.

Thus, four guiding questions were used, namely: based on the structure of the ICUs, what is your perception of the quality of palliative care provided? Based on your experience, how is patient care in palliative care systematized? What is your perception of the effect of your assistance on the palliative care patient’s health status? What is your perception of the effect of assistance from the multiprofessional team on the palliative care patient’s health status?

The interviews were recorded using a digital device and fully transcribed to ensure greater reliability on the data collected, with an average audio recording time of 15 minutes.

### Data Analysis and Treatment

To evaluate the data collected, the Content Analysis technique was used, in which data can be evaluated according to three types of approach: Conventional, Summative, and Direct^(13)^. In this study, we opted for the Direct analysis, in which, from the anchored theoretical framework, the main concepts or variables are established as initial coding categories^(13)^.

Following full reading of the material, the content was interpreted and organized in the light of Donabedian’s theory, to highlight the characteristics of the quality of care provided by a health service, and the answers were grouped in pre- established categories: structure (characteristics of the place where care is provided), process (organization and implementation of assistance), and outcome (effect of the care provided to the individual’s health status)^(8)^. Then, a new reading was performed, electing the sentences with greater emphasis by the participants, from which five subcategories emerged. As a way of ensuring data validation, as well as of reducing biases resulting from a single perspective, this step was performed by two researchers.

### Ethical Aspects

The study was approved by the Research Ethics Committee of the Universidade Estadual do Sudoeste da Bahia, confirmed by opinion no. 4.130.461, of the year 2020. Research participants were previously informed about the objective and methodological proposal of the study, and later signed the Free and Informed Consent Form (FICF). The interviews were identified by the first letter referring to the participant’s professional category, followed by the number corresponding to their interview, for example: nurses (N1); nursing technicians (T2); physical therapists (PT3); pharmacists (PA4); physicians (Ph5); psychologists (P8); nutritionists (Nt9).

## RESULTS

Thirty-five professionals who provided care to PC patients in ICUs, aged between 21 and 56 years old, participated in the study, with a mean of 35.46 years (± 8.7). Regarding sex, 28 (80%) of the participants were female. Regarding the professional category, nine (25.7%) were nursing technicians, seven (20%) were nurses, six (17.1%) were physical therapists, four (11.4%) were physicians, four (11.4%) psychologists, three (8.6%) nutritionists, and two (5.7%) pharmacists.

As for the length of time working in the ICU, it ranged between five months and 14 years, with a mean of 48.34 months (± 52.9), and 24 (68.6%) professionals reported up to five years of experience in the department. With regard to professional training related to the PC area, no participant had undergone training courses and 13 (37.1%) had participated in some updating activity in the aforementioned area.

**Chart 1.**
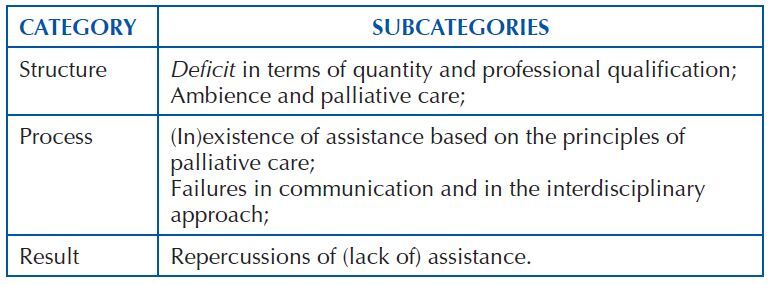
Categories and subcategories emerged from the study – Jequié, BA, Brazil, 2020.

Through the analysis of the researched content, as well as considering Donabedian’s theoretical framework, three categories were pre-established: *structure*, *process* and *outcome.* Of these, five subcategories emerged, as exposed in Chart 1.

## CATEGORY 1: STRUCTURE

The first category, called *structure*, refers to the professionals’ statements about the institutional characteristics required for care to occur, passing through ideas ranging from organizational aspects, such as human resources, to physical attributes of the institution and their implications for qualified care, expressed in the subcategories *Deficit in number of workers and professional qualification* and *Ambience and palliative care*.

### Subcategory: Deficit in Number of Workers and in Professional Qualification

In this subcategory, the concerns of the study participants pointed to the need for a greater number of professionals to work in the ICUs, as well as training in the area of PC: *In terms of the number of professionals, I think there are several categories (…) I think we have insufficient number of professionals…. Right? So, sometimes you have a very serious patient, you have a tight sizing in the ICU, and this greatly compromises the provision of palliative care for a patient, you know? (…)* (FA7).

*(…) We have a nutritionist in more than five hospital wards, so it is impossible for her to be present inside this ICU, (…) so (…) when you have this rotation (multiprofessional rounds) (…), neither the psychologist nor the nutritionist can be present because there is a lower number of professionals for a greater demand from patients (N28).*

Furthermore, the lack of professional qualifications in the PC area directly interferes with both the efficiency and the use of available resources for this approach, negatively impacting the care provided: *As for the structure and what it offers in terms of equipment, technology, I think it’s the ideal. But I think the most important thing is the human factor to deal with it, and even in my opinion, they are a little unprepared, regarding the use of the equipment that the institution offers to provide care to these palliative patients* (T31).

*Based on the physical structure, I think the hospital is able to perform a good job in this regard, but based on the technical structure, I think it needs more qualification (…) more qualified professionals (…) I believe that the technical team needs to evolve, and still a lot, in all aspects* (P33).

Furthermore, the respondents consider it important to have a team with theoretical and practical training in PC, allowing guidance of care for these patients: *In fact, the hospital is still a novice in terms of palliative care, you know? We don’t have an experienced team in palliative care to guide, so it’s a lot… it’s… still vague in the palliative care issue (…)* (F17).

*So, I still don’t see a team dedicated to this, sometimes I see that it’s wrong of us to do some things, to have some behaviors that (…) could be treated in another way (…) There should be a team dedicated to palliation in the hospital, not just an on-call team (…)* (T29).

### Subcategory: Ambience and Palliative Care

Participants express difficulties related to the physical structure and the availability of adequate material resources in the ICUs, echoing the humanization and comfort of the work process among professionals, patients, and family members: *(…) We have ICUs that do not offer anything, such as a television (…) there are ICUs with no windows (…) there are ICUs where the patient does not see the sun, does not see the moon… the family member is not comfortable there. If you have to talk to a family member (…) in some ICUs here, in most places, the patient’s report would be informed here (in a corridor). (…) There is no room for you to meet the psychologist, the social worker (…)* (F17).

*(…) It is (…) if we move to the issue of humanization, of giving the patient a more pleasant environment (…). ICU-03, for example, is a totally closed ICU, which does not have daylight, which does not have many things, you know? For the patient to feel more embraced* (F16).

Likewise, the professionals ratify the need for a structured unit for PC patients, since the ICUs do not provide a welcoming environment, ending up distancing the patient from his/her family, as explained in the following statements: *(…) having a place for these patients in palliative care, with a little more flexibility, to be close to the family… to have more visits (…) something like that (…) Because here inside the ICU there is not much condition, but if we had an appropriate place just for them to have more contact, it would be very good* (T9).

*(…) If it’s palliative care, I think this patient wouldn’t need to stay in an ICU. I think this patient’s situation should be optimized and he/she should be transferred to a palliative care unit (…) and there should be a unit just for palliative care (…) so he could be with his/her family* (F15).

## CATEGORY 2: PROCESS

The category named *process* reveals, in the narratives presented, weaknesses in the operationalization of care, from the lack of protocols guiding this practice to the failure in communication among the team, revealed by the statements organized in the subcategories: (*In)existence of assistance based on the principles of palliative care* and *Failures in communication and in the interdisciplinary approach*.

### Subcategory: (In)Existence of Assistance Based on the Principles of Palliative Care

This category is based on the perception that there is disorganization of care flows for the eligibility of patients in PC: *I believe that behavior, concepts standardization is required, so that we can work in a joint and objective way. Because it’s still a little vague, we still don’t have a standard definition of how to start, and for which patients this type of situation is elective. So it would really be to formalize a standard here within our ICU* (M13).

*I think (…) a protocol is required, on how to really act and how to define where to leave this patient. There are patients here, who were intubated with… who had already talked to the family about being in palliative care (…)* (F16).

This fact is present in the medical team’s decision-making for the eligibility of these patients, as reported in the following interviews: *(…) it usually depends a lot on the family to give an answer, I usually feel that on the medical side. After the family says no, that the person did not want to be intubated in life…. then they feel confident for not investing more… I think the protocol is more based on what the family member says* (E1).

*The difficulty is that some medical professionals understand that it is a medical diagnosis and not a family opinion, you know? (…) some medical professionals are afraid of diagnosing the patient and telling the family that that patient has a limit of therapeutic effort and that that patient will start palliative care* (E14).

### Subcategory: Failures in Communication and in the Interdisciplinary Approach

Professionals also consider that there are gaps in communication between the team and assistance centered on the medical decision, without the participation of other members, this being a complicating factor for the planning and continuity of care provided, leading to fragmentation in the interdisciplinary approach:

*The lack of communication itself. The lack of communication on how to act with them, you know? Because the patient often goes into palliative care, but then, another professional comes, and takes him/her from it, and then, there is lack of communication for the care to be better.* (T12).

*It doesn’t exist, you know? This multi-professional issue. What I’ve seen a lot in ICU-03 (…): The doctor who was responsible for the decision, without doing “diarismo” (rounds with the participation of the multiprofessional team) (…) skipped “diarismo” and put there “Palliative care” (…) The diarist doctor working on the next day didn’t agree with that palliative care, so he didn’t write it in the chart. The doctor working the other day didn’t pass the bulletin emphasizing the issue of palliative care, right? (…) the doctor took HbA1c out of schedule, took out the question of blood gas analysis and some medications. The other one, on the other day, put it all over again…* (P8)

## CATEGORY 3: RESULT

### Subcategory: Repercussions of (Lack of) Assistance

As for the category entitled *outcome*, this is presented as the outcome of the categories *structure* and *process*, in which the gaps observed reverberated in an impairment to efficacy and effectiveness of the therapeutic proposal, reflecting on the patient’s safety and quality of life, giving rise to the subcategory *Repercussions of (lack of) assistance*, identified through the units of analysis:

*(…) we have palliative care patients who use pain medication, we have palliative care patients who do not use pain medication. In fact, we have a disorganization of assistance in relation to palliative care, because this is at the mercy of the person on duty that day, who is also not trained, you know? They are unable to do that* (E32).

*(…) the patient is poorly assisted, especially in terms of bathing (…) it is (…) thinks that because the patient is in palliative care he/she cannot have it, you know? Not a complete bath (…) just intimate area and oral hygiene (…) so there is certain resistance. There is even a line, which is something I wanted to disqualify, which is: “Ah, but isn’t he/she in palliation? But won’t the patient die?”* (E14).

## DISCUSSION

The findings evidenced in this study, through the indicators of structure, process, and outcome, allow a global understanding of the institutional aspects that lead to impairment of quality palliative care, given that these implications are multifactorial, not limited to the outcome alone at the end of care. In this context, its findings point to the need for readjustment in terms of number of workers and professional qualification, improvements in the physical structure and in the work process as a way of reducing disparities that compromise the quality of care.

Thus, the structure is understood as the characteristics of the place where care is provided, taking the physical, human, and financial resources into account; the process refers to the organization and implementation of assistance, while the result is the dimension that corresponds to the effect of the implemented therapy on the individual’s health status^(8)^. Therefore, the quality of care is evidenced after considering the balance between the positive and negative aspects expected in all these stages of evaluation. The final result, therefore, derives from the sum of scientific knowledge, available health technologies, and their applicability in patient care^(14)^.

Therefore, the structural weaknesses revealed in this study point to the *deficit* in terms of number of workers and professional qualification, as well as to weaknesses in the ICU environment for the applicability of PC, which may directly affect the assistance to the individual. A study carried out with 34 professionals from an intensive care team highlights that team undersizing predisposes to a greater occurrence of adverse events during health care, due to overload and precarious work conditions to which workers are exposed^(15)^.

In this regard, it is necessary to establish legal parameters to adequately dimension the teams working in highly complex environments, taking the requirements of the National Health Surveillance Agency (ANVISA) and the standards of the Councils of Professional Classes into account, promoting, in this way, the reduction of risks and unnecessary harm to the patient’s health^(16)^. However, it is observed that there are no criteria in the literature for the adequate dimensioning of professionals working with patients in PC, which should be explored, since the profile of patients eligible for this therapy requires biopsychosocial and spiritual care to the detriment of their clinical condition, requiring meticulous assistance for the prevention and adequate control of symptoms, in view of the imminent suffering they present, in life-threatening conditions.

Regarding the professional qualification profile in the PC area, a survey carried out with a multidisciplinary ICU team revealed the existence of *deficit* in adequate training to deal with PC patients, especially in the care of terminally ill patients^(17)^. This fact coincides with the data of this study, and needs to be debated institutionally, since the minimum level of complexity for carrying out this practice in non-specialized environments requires basic training of twenty to forty hours, as recommended by the WHO^(18)^.

This training precariousness results in limiting practices, which affect the individual’s quality of life, as health professionals are not adequately trained to effectively prevent and manage the symptoms and signs arising from multidimensional suffering. Therefore, it is essential to train human resources for the development of essential skills for palliative care, so that good practices in health care, combined with safe care for patients and their families^(19)^, are carried out.

With regard to the ambience of ICUs for the promotion of PC, the findings of this study are similar to the results of an integrative review, which reveals that this scenario is associated with negative factors, such as family distancing, lack of privacy, insufficient physical space, poor lighting and unalterable stressors, which are required in health care, such as continuous monitoring and use of devices^(20)^. This way, alternatives must be considered for the construction of environments that provide comfort and humanization for the care process^(21)^, such as physical adaptations, possibility of extended visits, spiritual support, and embracement of patients and their families.

In this context, the National Academy of Palliative Care^(22)^ proposes ways to guide the implementation of this approach in a hospital environment, considering three possibilities: through a Palliative Care Unit, through a consulting/mobile team or through an Itinerant Team. The *Palliative Care Unit* has specific beds for this type of care, being operated by a team trained for this service; the *Consulting or mobile team* guides behaviors when signaled by the atending physician, but does not operationalize care; the *Itinerant Team,* on its turn, performs the practice of care when signaled by the attending physician, with the latter having the responsibility of making the decision to continue monitoring the case together or not.

With regard to the work process to operationalize this approach, a lack of assistance based on PC and failures in communication and interdisciplinary approach are observed. Such aspects are observed in hospital units that do not encompass PC as a care philosophy and do not have palliative teams to guide these actions. Furthermore, in these places, barriers to the dissemination of this practice are still observed, such as the absence of care protocols, lack of professional training, and few bioethical discussions among the team^(10)^.

These organizational gaps make the provision of care centered on personal experiences, focused on the disease in relation to the patient, leading to the late onset of PC and, consequently, the prolongation of multidimensional suffering. Therefore, it is necessary to establish guidelines for the effectiveness of this therapeutic approach, taking the singularities of the subjects involved into account.

In the meantime, the Constitution of the Federative Republic of Brazil^(23)^, in its first and fifth articles, concerns, respectively, the right to the dignity of life and the non-submission to torture and inhumane treatments. These prerogatives support the philosophical and technical principles of palliative care, guaranteeing them as a legal right for patients and their families.

Therefore, the Ministry of Health^(24)^ published Resolution No. 41, of October 31, 2018, which deals with the organizational guidelines of PC in Brazil, establishing the beginning of this approach as early as possible, concomitantly with disease- modifying treatment, preventing and promoting the relief of suffering within the biopsychosociospiritual scope, extending this care to family members and/or companions, free from diagnostic and therapeutic futility. Thus, death is understood as a natural process of living, ensuring empathic communication, with respect to the truth, providing the patient’s autonomy through an active life.

Furthermore, if these principles have been established since the medical diagnosis, they lead to a greater probability of the patient and his/her family understanding his/her clinical condition, creating a more effective bond with the team following them^(25)^. This relationship of reliability benefits the work process within professionals, patients, and family members. Moreover, the incorporation of PC at the end of life reduces the need for diagnostic and therapeutic interventions that no longer bring benefits to the patient, which, consequently, implies a reduction in care costs^(26)^.

Regarding the interlocution of the multidisciplinary team reported in this study, the failures in communication observed are considered one of the main factors leading to the impairment of the quality of health care and patient safety^(27)^. According to a study carried out with 44 health professionals, in three hospitals in the city of Porto Alegre, Rio Grande do Sul, Brazil, it was possible to observe the presence of failures in the conveying of information among the team about the alterations and/or continuity of the therapeutic plan, as well as weaknesses in the patient’s history and clinical evaluation records, contributing to the increase in the occurrence of adverse events^(28)^.

In this context, the *deficits* found in the communication process create difficulties for the participation of the multidisciplinary team in the conduction of PC. Consequently, decisions become uniprofessional and the patient’s and family’s multidimensions cannot be contemplated. It is necessary to encourage the democratic participation of all team members and, above all, of the patient and family^(29)^.

Given the institutional characteristics observed in this research, the category *outcome* denoted the effect of the aspects studied in the provision of care to patients eligible for PC, directly impacting the patient’s life. As analyzed, the patient’s quality of life and safety are impaired, as in addition to the ineffective management of symptoms and signs, they become vulnerable to incidents and damage caused by fragile care.

Thus, a study carried out in the *National Health Service*, in England, during a period of 12 years, through the analysis of reports of serious incidents that require investigation involving patients in PC, found 475 reports of adverse events. Of these, 266 were related to pressure injuries, 91 to medication errors, 46 to falls, 21 to health care-related infections, and 35 to other circumstances. The main causes highlighted were the lack of professional experience in PC, *deficit* of physical resources, and poor service management^(30)^.

The occurrence of adverse events contributes to the perpetuation of the illness process, leading to extended hospital stay, as well as increase in care costs. Through these damages, the degree of care dependence is increased, enhancing work overload of the multiprofessional team.

The findings of this study point to the urgency of establishing care quality criteria in the approach to PC patients in the ICU setting, correlating structural and work process factors, which directly reverberates in the results of the care provided. In addition, they provide subsidies for planning, monitoring, and evaluation of managers and health professionals for the establishment of evidence-based practices.

As for its limitations, it should be considered that it reflects the reality of only one health unit located in an inland area of the State of Bahia, Brazil, where there was no implementation of a team and/or unit specialized in PC. Therefore, the importance of further studies on the theme is highlighted to promote reflections on the quality of health services assiting patients in PC.

## CONCLUSION

The study allowed us to understand the institutional weaknesses on the quality of health care provided to patients eligible for PC, in the ICU setting. The characteristics evidenced highlight the need to readjust the structural demands and the work process, as they are divergent from the philosophical and technical principles of PC.

However, for this philosophy to be propagated in the ICUs, adequate investment in the number of workers, in professional training, physical structure, implementation of norms, routines and protocols is required, as a way of subsidizing the actions provided to these patients. In addition, evaluation indicators should be instituted, making it easier for health professionals to gradually and systematically analyze the evolution of the quality of care, aiming to determine the efficiency and effectiveness of the care provided, besides proposing adjustment strategies for non-satisfactory aspects. satisfactory.

Therefore, the co-participation of managers, multidisciplinary team, patients, and family members is of paramount importance for the final result of this type of care, since these gaps cannot be filled without collective involvement.

## ASSOCIATE EDITOR

Thiago da Silva Domingos
